# Clinical and epidemiological aspects of coinfection with American tegumentary leishmaniasis and HIV/AIDS in South American countries: a systematic review

**DOI:** 10.1590/0037-8682-0128-2026

**Published:** 2026-10-16

**Authors:** Carolina Sad Navarro, Selma Costa de Sousa, Pedro Alves Soares Vaz de Castro, Mariângela Carneiro, Juliana Maria Trindade Bezerra

**Affiliations:** 1 Universidade Federal de Minas Gerais, Instituto de Ciências Biológicas, Programa de Pós-Graduação em Parasitologia, Belo Horizonte, MG, Brasil.; 2 Northwestern University, Feinberg School of Medicine, Department of Pathology, Chicago, IL, USA.; 3 Universidade Estadual do Maranhão, Programa de Pós-Graduação em Ciência Animal, São Luís, MA, Brasil.; 4 Universidade Estadual do Maranhão, Programa de Pós-Graduação em Biodiversidade, Ambiente e Saúde, Caxias, MA, Brasil.; 5 Universidade Estadual do Maranhão, Departamento de Química e Biologia, Campus Caxias, Caxias, MA, Brasil.

**Keywords:** American tegumentary leishmaniasis, HIV/AIDS, Epidemiology, South America

## Abstract

Leishmaniasis is one of the 10 leading neglected tropical diseases (NTDs), and cutaneous leishmaniasis is endemic in 19 countries in the Americas. This study aimed to systematically review the epidemiological and clinical characteristics, diagnostic methods, and outcomes associated with coinfection with American tegumentary leishmaniasis (ATL) and HIV in South American countries. This review was registered in the International Prospective of Systematic Reviews (PROSPERO; CRD42023138800). Searches were conducted in the following electronic scientific databases: the *Biblioteca Virtual em Saúde*, *Literatura Latino-americana e do Caribe em Ciências da Saúde*, SciVerse Scopus, the US National Library of Medicine (PubMed) and Web of Science, using subject headings from the *Descritores em Ciências da Saúde* and Medical Subject Headings in Portuguese, English and Spanish for studies published from 1987 to 2025. Two independent reviewers performed study selection. The methodological quality of the included studies was assessed using the Joanna Briggs Institute Critical Appraisal Checklists. Overall, 417 participants from 32 studies were included in this systematic review. Argentina, Bolivia, Brazil, Colombia, Ecuador, Paraguay and Peru reported concurrent ATL and HIV. In 29 studies (90.6%), the diagnosis was established based solely on clinical criteria. Diagnostic methods for ATL included culture and the Montenegro skin test, whereas those for HIV included Enzyme-Linked Immunosorbent Assay, Western blotting, and indirect immunofluorescence. Approximately 68% of the analyzed data were derived from case reports, limiting the overall level of evidence. Strengthening integrated surveillance is needed to improve the clinical management of patients with ATL-HIV coinfection and expand access to health services.

## INTRODUCTION

American tegumentary leishmaniasis (ATL) is transmitted through the bite of infected female sand flies of the genus *Lutzomyia* (Diptera: Psychodidae) and can manifest in three clinical forms: cutaneous (CL), mucosal (ML), and diffuse (DL). Leishmaniasis is among the 10 leading neglected tropical diseases (NTDs), affecting more than 12 million people worldwide; its cutaneous form is endemic in 19 countries in the Americas^1^. In 2024, three South American countries accounted for 82% of CL cases: Brazil, Colombia and Peru^1^. In Brazil, the main causative agents include *L. (Viannia) braziliensis*, *L. (Viannia) guyanensis*, and *L. (Leishmania) amazonensis* ^1^. 

In that same year, 9,810 cases of CL and ML were reported in Brazil, 6,002 in Colombia, and 5,323 in Peru. Brazil also reported the highest number of ML cases (636), followed by Peru (457) and Bolivia (267). Compared with 2023, the overall number of reported cases decreased by 14% in 2024. However, increases were observed in Colombia (22%), Peru (2%), Ecuador (13%), Suriname (95%), and Venezuela (8%). 

Human Immunodeficiency Virus (HIV) causes Acquired Immunodeficiency Syndrome (AIDS) disease and remains a major global public health concern. In 2024, approximately 40.8 million people were living with HIV worldwide, and HIV-related causes accounted for approximately 630,000 deaths^2^. In the Americas, 170,000 new HIV infection cases were reported in 2024^1^. Human Immunodeficiency Virus infection progressively depletes CD4^+^ T lymphocytes, impairing cell-mediated immunity and increasing susceptibility to opportunistic infections^3^.

In the Americas, 159 cases of ATL-HIV coinfection were reported in 2024. Of these, 132 (83%) were reported in Brazil^1^. Host and environmental factors may influence the coinfection. However, little is known about the clinical characteristics of ATL-HIV coinfection in South America, where the available evidence remains limited and fragmented, hindering a comprehensive understanding of its clinical manifestations, disease course, and potential complications^3^ ^,^ ^4^. 

Given this context, this systematic review aimed to characterize the epidemiological and clinical features, diagnostic methods, and outcomes of ATL-HIV coinfection in South America, providing a more comprehensive understanding of the magnitude and characteristics of this coinfection.

## METHODS

A systematic review was conducted to address the following question: “What are the epidemiological, clinical, and diagnostic methods and consequences of ATL and HIV coinfection in South American countries between 1987 and 2025?”

### Study design

This study was registered in the International Prospective Register of Systematic Reviews (PROSPERO) under the number CRD42023138800 (PROSPERO)^5^ and followed the recommendations of the Preferred Reporting Items for Systematic Reviews and Meta-Analyses (PRISMA)^6^.

### Eligibility criteria

Studies published in English, Portuguese, and Spanish that reported epidemiological and clinical characteristics, diagnostic methods, or outcomes of ATL-HIV coinfection in South American countries between 1987 and 2025 were eligible for inclusion. The searches were initially conducted in September 2023 and updated through August 2026 to identify newly published studies. Only full-text scientific articles with the following study designs were included: case reports, cross-sectional, case-control, case series, and cohort studies.

### Databases

Electronic databases of the *Biblioteca Virtual em Saúde*, *Literatura Latino-americana e do Caribe em Ciências da Saúde*, SciVerse Scopus, US National Library of Medicine (PubMed), and Web of Science were searched for relevant studies.

### Search Strategy

The descriptors were defined by consulting the *Descritores em Ciências da Saúde* and Medical Subject Headings databases and in Portuguese, English, and Spanish. The Boolean operators “AND” and “OR” were used, as well as quotation marks to facilitate the search for manuscripts.

The search strategy was based on combinations of terms either together or separately, in the three languages mentioned above: ("*Leishmaniasis*") OR (*Leishmaniases* OR *Leishmania Infection* OR *Leishmania Infections*) OR ("*Leishmaniasis*, *Diffuse Cutaneous*") OR (*Diffuse Cutaneous Leishmaniases* OR *Diffuse Cutaneous Leishmaniasis*) OR ("*Leishmaniasis*, *Mucocutaneous*") OR (*Mucocutaneous Leishmaniases* OR *Mucocutaneous Leishmaniasis*) OR ("*Leishmaniasis*, *Cutaneous*") OR (*Cutaneous Leishmaniasis* OR *Old World Leishmaniasis* OR *New World Leishmaniasis* OR *American Leishmaniasis*)” The bibliographic search was assisted by a professional librarian, tested and reproduced by two researchers. If consensus could not be reached regarding the bibliography, a third researcher was invited to resolve the impasse.

### Assessment of the methodological quality of the included studies

Case reports, cross-sectional, case-control, case series, and cohort studies included in the study were evaluated for methodological rigor using the Joanna Briggs Institute Critical Appraisal Checklist^7^.

### Study selection and data extraction

The search results were exported using the Rayyan (Qatar) application, through which duplicate articles were identified and removed from the database. Two independent reviewers examined the titles and abstracts of all manuscripts that met the inclusion criteria. Selected titles were read in full, and additional studies were excluded. When the two reviewers did not agree on the exclusion of a study, a third reviewer was asked to assist in the decision. Manuscripts that did not answer the question in the text, were not related to the study question, were not conducted in South America, or were not in scientific article format were excluded. Information was extracted from the full texts (selected studies). Data on authorship, year, objectives, results, discussion, and conclusion were extracted from the studies, in addition to: epidemiological characteristics (study period, study type, study area and population, sex, and age of patients); laboratory characteristics (immunological diagnostic methods, causative species, HIV detection test, and opportunistic infections); and finally, clinical and treatment characteristics (treatment and outcomes). Microsoft Word and Excel 2019 (Redmond, Washington, United States of America) were used to organize tables and graphs.

## RESULTS

### Analysis of studies

A total of 2,304 articles were identified, of which 895 duplicate studies were excluded, leaving 1,409 studies. After this phase, 1001 articles were excluded after reading the title, leaving 408 studies, of which 291 were excluded after reading the abstract. Afterwards, 117 studies were analyzed, of which 85 were excluded after reading the full text. At the end of this stage, 32 studies met the selection and eligibility criteria established for this systematic review (Figure 1).

**FIGURE 1:**
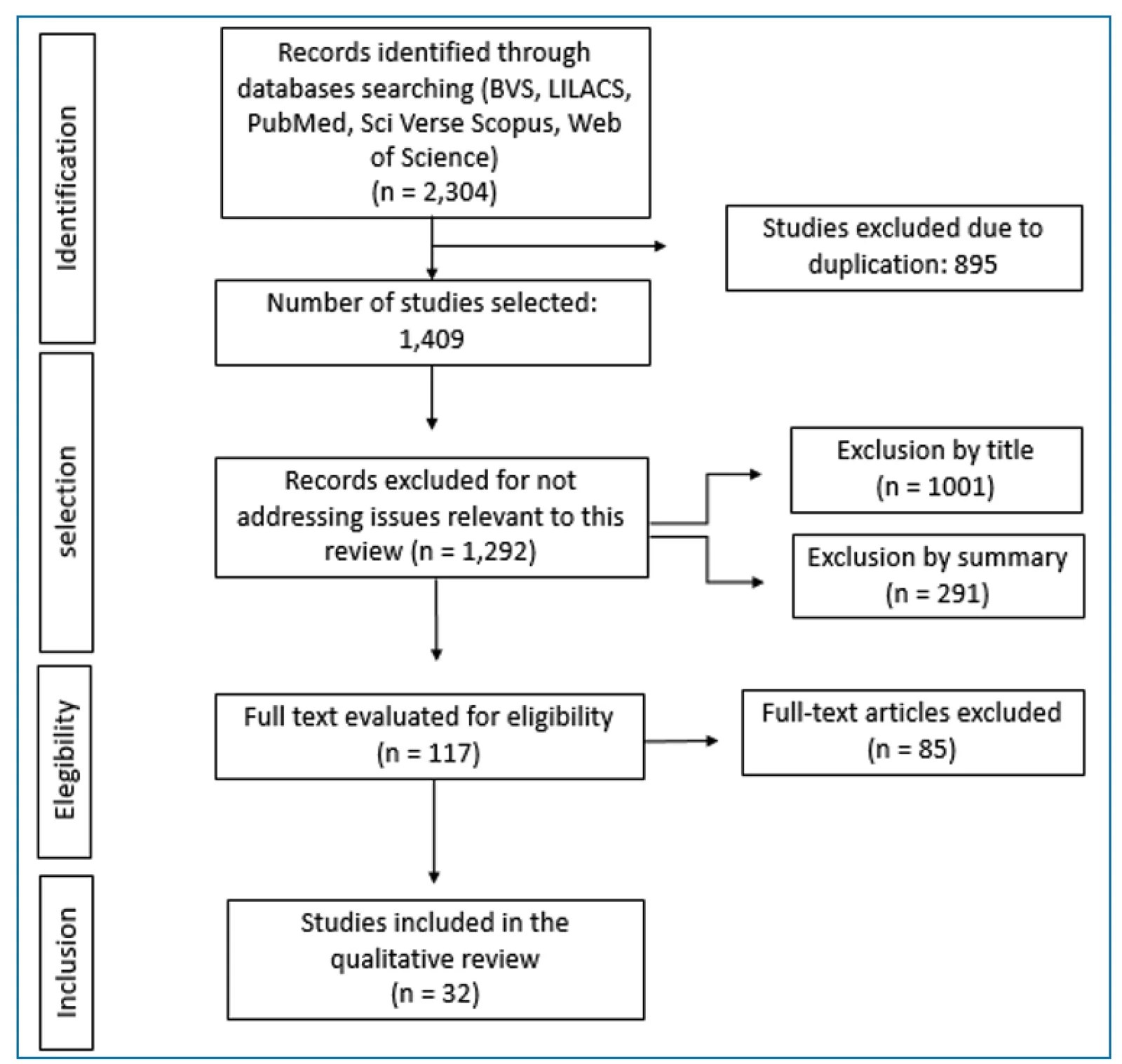
Flowchart of the studies included in the systematic review on coinfection with American tegumentary leishmaniasis and HIV/AIDS in South American countries.

The 32 studies demonstrated the epidemiological, clinical, and diagnostic methods and the outcomes of ATL-HIV coinfection in South American countries (Table 1). Among the selected studies, 21 (65.6%) were case reports, 4 (12.5%) cross-sectional studies, 3 (9.3%) case series, 3 (9.3%) case-control studies, and 1 (3.1%) a cohort study.

**TABLE 1:** Studies included in the systematic review, demonstrating the period of completion, region and design, on the coinfection of American tegumentary leishmaniasis and HIV/AIDS in South American countries.

Author(s)	Study period	Study region	Study design
Coura et al.^8^	1987	Brazil	Case report
Da-Cruz et al.^9^	1992	Brazil	Case report
Machado et al.^10^	1992	Brazil	Case report
Souza et al.^11^	1995 - 1996	Brazil	Case report
Rosatelli et al.^12^	1998	Brazil	Case report
Da-Cruz et al.^13^	1999	Brazil	Case report
Romero et al.^14^	2004	Argentina	Case report
Pérez et al.^15^	2006	Colombia	Case report
Soares et al.^16^	2006	Brazil	Case control
Chrusciak-Talhari et al.^17^	2009	Brazil	Case report
Torrico et al.^18^	2006	Bolivia	Case report
Santos-Oliveira et al.^19^	2010	Brazil	Case control
Castellano et al.^20^	2011	Brazil	Case report
Hozannah et al.^21^	2013	Brazil	Case report
Montenegro-Idrogo et al.^22^	2014	Peru	Case report
Calvopina et al.^23^	2017	Ecuador	Case report
Lyra et al.^24^	2019	Brazil	Case series
Soares et al.^25^	2020	Brazil	Case report
Araújo et al.^26^	2021	Brazil	Case report
Amato et al.^27^	2023	Brazil	Case report
Laverde-Sudupe et al.^28^	2025	Colombia	Case report
Da Silva et al.^29^	2014	Brazil	Case report
Guimarães et al.^30^	2015	Brazil	Cross sectional
Parmentier et al.^31^	2016	Paraguay	Case report
Durães et al.^32^	2023	Brazil	Case report
De Oliveira et al.^33^	2012	Brazil	Cross sectional
Amato et al.^34^	2009	Brazil	Cohort
Guerra et al.^35^	1999 - 2008	Brazil	Case series
Rodrigues et al.^36^	2011	Brazil	Cross sectional
Oliveira et al.^37^	2011	Brazil	Cross sectional
Gois et al.^38^	2014	Brazil	Case series
Couppie et al.^39^	2004	French Guiana	Case control

Of the 32 studies analyzed, 24 (75%) were conducted in Brazil, with 10 (41.7%) conducted in the state of Rio de Janeiro (Southeast region), 4 (16.7%) in Amazonas (North region), 4 (16.7%) in Bahia (Northeast region), 2 (8.3%) in São Paulo (Southeast region), 1 (4.2%) in Pernambuco (Northeast region), 1 (4.2%) in Mato Grosso do Sul (Midwest region), 1 (4.2%) in Paraná (South region), 1 (4.2%) in Maranhão (Northeast region), 1 (4.2%) in Goiás (Midwest region), and 1 (4.2%) in Alagoas (Northeast region). For the eight remaining studies, two (6.2%) were conducted in Colombia, while Argentina, Bolivia, Ecuador, French Guiana, Paraguay, and Peru each contributed one study, accounting for 3.1% of studies. Furthermore, the species *L. braziliensis* is the most prevalent in South American countries (Table 1 and Figure 2).

**FIGURE 2:**
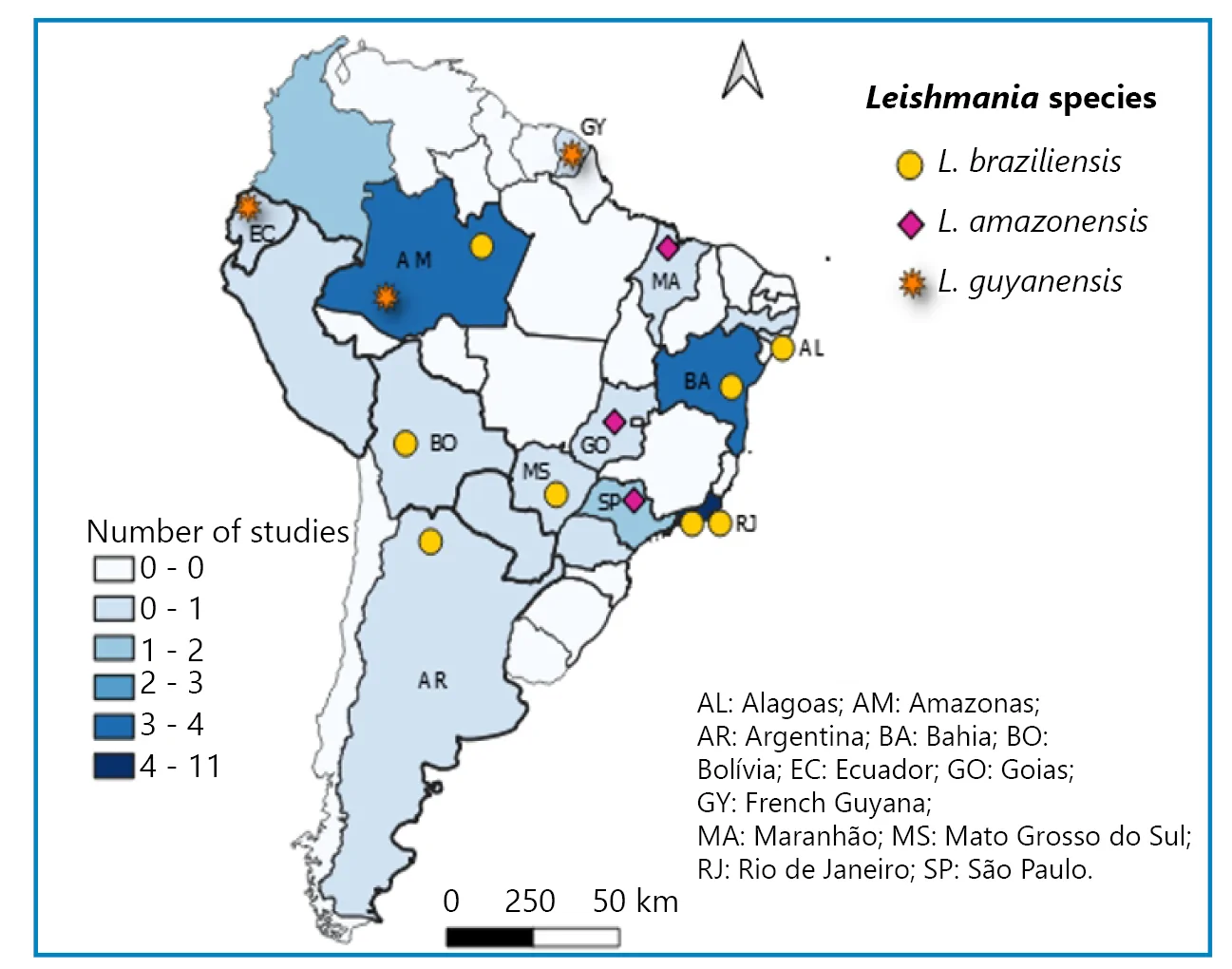
Distribution of studies published between 1987 and 2025 on the epidemiological and clinical of coinfection with American tegumentary leishmaniasis and HIV/AIDS in South American countries, according to the geographic area of ​​study, as well as the *Leishmania* species found in each state/country.

### Sociodemographic characterization of American tegumentary leishmaniasis and HIV/AIDS coinfection cases

Of the 32 studies, 21 reported that 65.6% of ATL cases and HIV/AIDS coinfection occurred in men^8^ ^-^ ^28^ and 12.5% in women^29^ ^-^ ^32^. In one study (3.1%), no information on the sex of the participants was provided^33^, while both sexes appeared in six (18.7%) studies^34^ ^-^ ^39^. Regarding the most affected age groups, the highest percentage of ATL-HIV coinfection cases (50% of cases) were observed in people aged between 20 and 40 years, followed by those between 40 and 60 years (29.4% of cases). None of the studies characterized data on education and income (Table 2).

**TABLE 2:** Sociodemographic characterization by sex and age group of individuals affected by coinfection between American tegumentary leishmaniasis and HIV/AIDS in South American countries.

Authors	Sex	Age	Sample
Coura et al.^8^	Men	57 years	1
Da-Cruz et al.^9^	Men	36 years	1
Machado et al.^10^	Men	36 years	1
Souza et al.^11^	Men	23 years	1
Rosatelli et al.^12^	Men	25 years	1
Da-Cruz et al.^13^	Men	53 years	1
Romero et al.^14^	Men	50 years	1
Pérez et al.^15^	Men	29 years	1
Soares et al.^16^	Men	Median age of participants: 32.3 years	15
Chrusciak-Talhari et al.^17^	Men	32 years	1
Torrico et al.^18^	Men	32 years	1
Santos-Oliveira et al.^19^	Men = 15	Median age of participants: 44 years	8
Castellano et al.^20^	Men	36 years	1
Hozannah et al.^21^	Men	33 years	1
Montenegro-Idrogo et al.^22^	Men	42 years	1
Calvopina et al.^23^	Men	32 years	1
Lyra et al.^24^	Men	Median age of participants: 43 years	5
Soares et al.^25^	Men	46 years	1
Araújo et al.^26^	Men	50 and 52 years	2
Amato et al.^27^	Men	52 years	1
Laverde-Sudupe et al.^28^	Men	24 years	1
Da Silva et al.^29^	Women	29 years	1
Guimarães et al.^30^	Women	31 years	1
Parmentier et al.^31^	Women	60 years	1
Durães et al.^32^	Women	2 years	1
De Oliveira et al.^33^	Not informed	Not informed	8
Amato et al.^34^	Men = 94 Women = 46	Median age of participants: 57.5 years	140
Guerra et al.^35^	Men = 14 Women = 1	Median age of participants: 36.2 years	15
Rodrigues et al.^36^	Men = 12 Women = 5	Median age of participants: 36 years	17
Oliveira et al.^37^	Men = 93 Women = 76	Not informed	169
Gois et al.^38^	Men = 5 Women = 3	Median age of participants: 39.7 years	8
Couppie et al.^39^	Men = 8 Women = 1	Median age of participants: 46 years	9

### Clinical characteristics and diagnostic methods of American tegumentary leishmaniasis and HIV/AIDS coinfection cases

Regarding the description of cases of ATL and HIV coinfection, 29 studies (90.6%) used clinical criteria to establish the diagnosis^8^ ^-^ ^15^ ^,^ ^17^ ^,^ ^18^ ^,^ ^20^ ^-^ ^35^ ^,^ ^37^ ^,^ ^38^ ^,^ ^39^. The tests reported in scientific studies for detecting ATL included culture and Montenegro intradermal reaction, with six studies (18.7%) using the first test^24^ ^,^ ^25^ ^,^ ^29^ ^,^ ^33^ ^,^ ^38^ ^,^ ^39^ and four (12.5%) using the second test^20^ ^,^ ^28^ ^,^ ^34^ ^,^ ^35^ (Table 3).

**TABLE 3:** Characterization of the studies included in the systematic review on the epidemiological, clinical, and diagnostic methods of American tegumentary leishmaniasis and HIV/AIDS coinfection in South American countries.

Authors	Confirmation criteria	ATL/HIV detection test	***Leishmania* species**	Symptoms	Lesion (local)	Recurrences/Reinfections	Opportunistic infections	Treatment	Outcome
Coura et al.^8^	Clinical criteria	ELISA, Indirect IF	*L. (Viannia) braziliensis*	Fever, weariness, weight loss, anorexic	Nodulus non ulcerated (thorax, lower limbs)	No	Not reported	Not reported	Death
Da-Cruz et al.^9^	Clinical criteria	Indirect IF	*L. (Viannia) braziliensis*	Weight loss	Laryngeal inflammatory to nasal septum	No	Oral candidiasis	Not reported	Not reported
Machado et al.^10^	Clinical criteria	ELISA	*L. (Viannia) braziliensis*	Fever	Ulcerative lesion and necrotising papules (soft palate, ears, penis and hand)	Yes	Oropharyngeal candidiasis	Glucantime	Death
Souza et al.^11^	Clinical criteria	ELISA, Indirect IF	*L.(Viannia) guyanensis*	Fever, diarrhea, sinusitis, scabies, dermatophytosis	Oedema and infiltration (nasal cavity, nostrils)	Yes	Oral candidiasis	Pentamidine	Healed
Rosatelli et al.^12^	Clinical criteria	ELISA, Western blot	*L. (Leishamania) amazonensis*	Weight loss, pneumonitis	Disseminated erythematous nodular (hand, arm, left foot)	No	Bilateral pneumonia with right pleural effusion	Glucantime	Death
Da-Cruz et al.^13^	Clinical criteria	ELISA, Indirect IF	*L. (Viannia) braziliensis*	Not reported	Ulcerative and papular (soft palate, mucous membrane, upper lip)	Yes	Not reported	Glucantime; Amphotericin B	Death
Romero et al.^14^	Clinical criteria	ELISA, Western blot	*L. (Viannia) braziliensis*	Fever, weight loss, diarrhea, dysphagia, vomiting, headache	Crusted papulovesicular lesions (nose, left leg and nasal septum)	Yes	Polyadenopathy, oral candidiasis	Glucantime; Amphotericin B	Death
Peréz et al.^15^	Clinical criteria	ELISA, Western blot	Not reported	Fever, weight loss, diarrhea, arthromyalgia, abdominal pain	Erythematous, macules and papules (palms and soles)	Yes	Not reported	Glucantime; Amphotericin B	Cured
Soares et al.^16^	Not reported	Not reported	Not reported	Not reported	Not reported	Not reported	Not reported	Not reported	Not reported
Chrusciak-Talhari et al.^17^	Clinical criteria	Not reported	*L. (Viannia) guyanensis*	Not reported	Not reported	No	toxoplasmosis	Glucantime; Amphotericin B; tenofovir; efavirenz; lamivudina	Coma
Torrico et al.^18^	Clinical criteria	ELISA, Western blot	*L. (Viannia) braziliensis*	Difficulty breathing, cough, sore throat, dysphagia, dysphonia	Maculo papulo nodular lesions (face, eyebrows, left ear lobe)	No	Pneumonia and bronchitis	Amphotericin B	Successful therapy
Santos-Oliveira et al.^19^	Not reported	Not reported	Not reported	Not reported	Not reported	No	Not reported	Not reported	Not reported
Castellano et al.^20^	Clinical criteria	Montenegro test; ELISA	*L. (Viannia) braziliensis*	Not reported	A perforation of the septum and a rounded scar on the right ankle	Yes	Not reported	Glucantime	Not reported
Hozannah et al.^21^	Clinical criteria	Not reported	Not reported	Not reported	Ulcerated lesion and infiltratated borders (malar region)	No	Not reported	Glucantime	Cured
Montenegro-Idrogo et al.^22^	Clinical criteria	Indirect IF	Not reported	Parietal headache	Papulo-ulcerative lesions (mucous membrane of nasal cavity)	Yes	Not reported	Zidovudine; Lamivudine; Lopinavir; Ritonavir	Alive
Calvopina et al.^23^	Clinical criteria	ELISA, Western blot	*L. (Viannia) guyanensis*	Not reported	Nodular maculopapular skin lesions that form ulcerative and crusted plaques (face, neck, ears)	Yes	Toxoplasmosis	Glucantime; Amphotericin B	Death
Lyra et al.^24^	Clinical criteria	Culture	*L. (Viannia) braziliensis*	Not reported	Genital lesions	No	Not reported	Glucantime; Amphotericin B	Not reported
Soares et al.^25^	Clinical criteria	Culture	*L. (Leishmania) amazonensis*	Pain on touch	Ulcerated lesion with raised edges; diffuse erythematous nodules (various parts of the body)	Yes	Acanthosis, Spongiosis	Glucantime; Amphotericin B	Cured
Araújo et al.^26^	Clinical criteria	ELISA, Indirect IF	*L. (Leishmania) amazonensis*	Not reported	Ulcerated and papular skin lesions (trunk, face, mucous membranes)	No	Paracoccidioidomycosis e tuberculosis	Glucantime; Amphotericin B; tenofovir; lamivudina	cured e Follow-up
Amato et al.^27^	Clinical criteria	Not reported	*L. (Viannia) braziliensis*	Weight loss, odynophagia	Ulcerated lesion with a granular appearance (nasal mucosa	No	Oral candidiasis	Amphotericin B	Healed
Laverde-Sudupe et al.^28^	Clinical criteria	Montenegro test	Not reported	Not reported	Erythematous ulcers with irregular edges (testicular, bilateral inguinal and anal regions)	No	Toxoplasmosis, herpes e tuberculosis	Dolutegravir; Rifampicina; Tenofir; Amphotericin B	Healed and Significant improvement
Da Silva et al.^29^	Clinical criteria	Culture	*L. (Viannia) braziliensis*	High fever, weight loss, cough, night sweats	Painless erythematous lesions; these progressed to scaly, itchy papules (lower limbs)	No	Lymphadenopath Pulmonary tuberculosis, toxoplasmosis	Glucantime; zidovudina; lamivudina; efavirenz	Cured
Guimarães et al.^30^	Clinical criteria	ELISA	*L. (Viannia) braziliensis*	Not reported	Ulcer (lower back and oral mucosa)	Yes	Not reported	Glucantime; Amphotericin B	Cured
Parmentier et al.^31^	Clinical criteria	Not reported	*L. (Viannia) braziliensi*s	Rhinitis	Ulcers with raised, erythematous edges (nasal mucosa)	No	Not reported	Amphotericin B; tenofovir; efavirenz	Cured
Durães et al.^32^	Clinical criteria	Not reported	*L. (Viannia) braziliensis*	Fever	Painful papules and vegetative ulcers	No	Osteomyelitis	Amphotericin B deoxycholate	cured
De Oliveira et al.^33^	Clinical criteria	Culture, ELISA, Western blot	*L. (Viannia) braziliensis*	Not reported	Lesions of the nasal mucosa; skin and mucosal lesions	Yes	Not reported	Glucantime; Amphotericin B;	Not reported
Amato et al.^34^	Clinical criteria	Montenegro test; ELISA, Indirect IF	Not reported	Not reported	Granulomatous-appearing edema or erosion (palatal mucosa)	Yes	Not reported	Glucantime,Pentamidine; Amphotericin B	Cured and not cured
Guerra et al.^35^	Clinical criteria	Montenegro test; ELISA, Indirect IF	*L. (Viannia) guyanensis;* *L. (Viannia) braziliensis*	Not reported	Perforation of the septum	Yes	Herpes zoster, Cerebral toxoplasmosis, candidíasis, Cryptococcosis, Lymphadenopathy	Glucantime; Pentamidine; Amphotericin B	Death and cured
Rodrigues et al.^36^	Not reported	Not reported	Not reported	Not reported	Not reported	Not reported	Not reported	Not reported	Not reported
Oliveira et al.^37^	Clinical criteria	Not reported	*L. (Viannia) braziliensis*	Not reported	Not reported	Not reported	Toxoplasmosis, candidíasis, herpes zoster, tuberculosis	Tenofovir; ritonavir; atazanavir	Not reported
Gois et al.^38^	Clinical criteria	Culture	Not reported	Not reported	Not reported	Not reported	Not reported	Not reported	Cured and not cured
Couppié et al.^39^	Clinical criteria	Culture, ELISA, Western blot	*L. (Viannia) guyanensis*	Not reported	Ulcers, papular lesions and non-ulcerated papular plaques	Yes	Not reported	Glucantime; Pentamidine	Death and cured

Elisa: Enzyme-Linked Immunosorbent Assay; Indirect IF: indirect immunofluorescence.

To confirm HIV, the tests used were Enzyme-Linked Immunosorbent Assay, Western blotting, and indirect immunofluorescence. The first was cited in 16 (50%) studies^8^ ^,^ ^10^ ^-^ ^15^ ^,^ ^18^ ^,^ ^20^ ^,^ ^23^ ^,^ ^26^ ^,^ ^30^ ^,^ ^33^ ^-^ ^35^ ^,^ ^39^, the second in 7 (21.8%)^12^ ^,^ ^14^ ^,^ ^15^ ^,^ ^18^ ^,^ ^23^ ^,^ ^33^ ^,^ ^39^, and the third in 8 (25%)^8^ ^,^ ^9^ ^,^ ^11^ ^,^ ^13^ ^,^ ^22^ ^,^ ^26^ ^,^ ^34^ ^,^ ^35^. In nine studies (28.1%), the tests used to detect ATL and HIV coinfection were not described^16^ ^,^ ^17^ ^,^ ^19^ ^,^ ^21^ ^,^ ^27^ ^,^ ^31^ ^,^ ^32^ ^,^ ^36^ ^,^ ^37^, and in eight (25%)^9^ ^,^ ^16^ ^,^ ^19^ ^,^ ^20^ ^,^ ^24^ ^,^ ^33^ ^,^ ^36^ ^,^ ^37^ the outcomes of ATL and HIV coinfection were not mentioned (Table 3). 

Regarding the causative protozoan species *L. (Viannia) braziliensis* was the most frequently identified species reported in 16 studies (50%)^8^ ^-^ ^10^ ^,^ ^13^ ^,^ ^14^ ^,^ ^18^ ^,^ ^20^ ^,^ ^24^ ^,^ ^27^ ^,^ ^29^ ^-^ ^33^ ^,^ ^35^ ^,^ ^37^, followed by *L. (Leishmania) amazonensis* reported in 3 (9.7%)^12^ ^,^ ^25^ ^,^ ^26^, and *L. (Viannia.) guyanensis* reported in 5 (15.6%)^11^ ^,^ ^17^ ^,^ ^23^ ^,^ ^35^ ^,^ ^39^ (Table 3).

Symptoms were described in 14 (43.7%) out of 32 studies^8^ ^-^ ^12^ ^,^ ^14^ ^,^ ^15^ ^,^ ^18^ ^,^ ^22^ ^,^ ^25^ ^,^ ^27^ ^,^ ^29^ ^,^ ^31^ ^,^ ^32^_._ Of these, fever was present in seven (50%)^8^ ^,^ ^10^ ^,^ ^11^ ^,^ ^14^ ^,^ ^15^ ^,^ ^29^ ^,^ ^32^, as well as the weight loss^8^ ^,^ ^9^ ^,^ ^12^ ^,^ ^14^ ^,^ ^15^ ^,^ ^27^ ^,^ ^29^. Three studies (21.4%) reported diarrhea in infected patients^11^ ^,^ ^14^ ^,^ ^15^. Headache^14^ ^,^ ^22^, cough^18^ ^,^ ^29^, dysphagia^14^ ^,^ ^18^, abdominal pain^15^ ^,^ ^25^, and sore throat^18^ ^,^ ^27^ were reported in two (14.3%) studies each. Other symptoms, such as sinusitis^11^, vomiting^14^, pneumonitis^12^, rhinitis^31^, respiratory distress^18^, dysphonia^18^, night sweats^29^, arthromyalgia^15^, and fatigue^8^, were present in one study each, with an individual percentage of 7.1% (Table 3).

Lesions and the areas affected by ATL were described in 26 studies (81.2%)^8^ ^-^ ^15^ ^,^ ^18^ ^,^ ^20^ ^-^ ^35^ ^,^ ^39^ (Table 3). Furthermore, 14 studies (43.7%) reported recurrence of this condition^10^ ^,^ ^11^ ^,^ ^13^ ^-^ ^15^ ^,^ ^20^ ^,^ ^22^ ^,^ ^23^ ^,^ ^25^ ^,^ ^30^ ^,^ ^33^ ^-^ ^35^ ^,^ ^39^ (Table 3).

In addition, 16 (50%) studies described opportunistic infections^9^ ^,^ ^10^ ^,^ ^11^ ^,^ ^12^ ^,^ ^14^ ^,^ ^17^ ^,^ ^18^ ^,^ ^23^ ^,^ ^25^ ^-^ ^29^ ^,^ ^32^ ^,^ ^35^ ^,^ ^37^. Of these, six (37.5%)^17^ ^,^ ^23^ ^,^ ^28^ ^,^ ^29^ ^,^ ^35^ ^,^ ^37^ reported toxoplasmosis or cerebral toxoplasmosis, seven (43.7%) reported that affected patients had candidiasis or oral candidiasis^9^ ^,^ ^10^ ^,^ ^11^ ^,^ ^14^ ^,^ ^27^ ^,^ ^35^ ^,^ ^37^, four (25%) reported tuberculosis^26^ ^,^ ^28^ ^,^ ^29^ ^,^ ^37^, three (18.7%) herpes zoster^28^ ^,^ ^35^ ^,^ ^37^, two (12.5%) both bacterial (*Streptococcus pneumoniae*)^12^ and fungal (*Pneumocystis carinii*)^18^ pneumonia; and cryptococcosis^35^, osteomyelitis^32^, acanthosis^25^, spongiosis^25^, bronchitis^18^, and paracoccidioidomycosis^26^ were reported in one study (6.2%) each (Table 3).

Regarding treatment, 26 studies (81.2%) mentioned the medications used to treat ATL-HIV coinfection. Of these, 18 (56.2%) used pentavalent antimony (Glucantime)^10^ ^,^ ^12^ ^-^ ^15^ ^,^ ^17^ ^,^ ^20^ ^,^ ^21^ ^,^ ^23^ ^-^ ^26^ ^,^ ^29^ ^,^ ^30^ ^,^ ^33^ ^-^ ^35^ ^,^ ^39^; 17 (53.1%) Amphotericin (B, liposomal or deoxycholate)^13^ ^-^ ^15^ ^,^ ^17^ ^,^ ^18^ ^,^ ^23^ ^-^ ^28^ ^,^ ^30^ ^-^ ^35^ ^;^ and 4 (12.5%) pentamidine^11^ ^,^ ^34^ ^,^ ^35^ ^,^ ^39^ to treat ATL infection. 

To treat AIDS, five studies (15.6%) cited the use of tenofovir^17^ ^,^ ^26^ ^,^ ^28^ ^,^ ^31^ ^,^ ^37^; four (12,5%) lamivudine^17^ ^,^ ^22^ ^,^ ^29^ ^,^ ^26^; three (9.3%) ritonavir^21^ ^,^ ^22^ ^,^ ^37^; three (9.3%) efavirenz^17^ ^,^ ^29^ ^,^ ^31^; two (6.2%) zidovudine^22^ ^,^ ^29^; and one (3.1%) lopinavir^22^. In six (18.7%) studies, no treatments for either disease were mentioned^8^ ^,^ ^9^ ^,^ ^16^ ^,^ ^19^ ^,^ ^36^ ^,^ ^38^ (Table 3).

Regarding the outcomes, eight studies reported death (25%)^8^ ^,^ ^10^ ^,^ ^12^ ^-^ ^14^ ^,^ ^23^ ^,^ ^35^ ^,^ ^39^ in men. The age group most affected by deaths was individuals aged between 50 and 60 years (50%) (Table 3).

### Quality analysis

The quality analysis of the case studies identified only one article with a high risk of bias^9^. The remaining studies were divided into 17 moderate-risk studies^8^ ^,^ ^10^ ^,^ ^11^ ^,^ ^13^ ^,^ ^15^ ^,^ ^17^ ^,^ ^18^ ^,^ ^20^ ^-^ ^22^ ^,^ ^25^ ^,^ ^28^ ^,^ ^29^ ^,^ ^31^ ^,^ ^32^ and 3 low-risk studies^12^ ^,^ ^23^ ^,^ ^26^ (Supplementary Material Table 1). The cross-sectional studies included three moderate-risk studies^30^ ^,^ ^36^ ^,^ ^37^ and one low-risk study^33^ (Supplementary Material Table 2). Of the case series studies, three were low-risk^24^ ^,^ ^35^ ^,^ ^38^ (Supplementary Material Table 3). In the case-control studies, the three studies presented a low risk of bias^16^ ^,^ ^19^ ^,^ ^39^ (Supplementary Material Table 4). One study presented a low-risk^34^ for the cohort design (Supplementary Material Table 5).

## DISCUSSION

This systematic review examined the epidemiological and clinical characteristics, diagnostic methods, and outcomes in individuals with ATL-HIV coinfection in South America. American tegumentary leishmanasis and HIV coinfection represents an important public health concern because of high relevance, given its high severity and expansion in various regions of the world. The 32 included studies were published between 1987 and 2025, and, to our knowledge, this is the first systematic review to synthesize evidence on ATL-HIV coinfection in South America during this period. Brazil accounted for the largest number of included studies, with 24 publications (81.8%). 

Most individuals with ATL-HIV coinfection were men. Several sociodemographic and behavioral factors may contribute to this predominance; however, the available evidence does not allow observed sex distribution to be attributed to a specific factor^40^ ^.^

This systematic review also found that most reported cases of ATL-HIV coinfection occurred among adults aged 20-60 years. Previous studies of ATL have similarly reported a predominance of cases among adults aged 20-49 or 20-59 years, which may reflect greater occupational, recreational, or other exposure to environments where sand fly transmission occurs^41^ ^,^ ^42^. Consistent with this explanation, a study of ATL cases across 15 European centers found that cases frequently occurred among active travelers, including young adults, military personnel, and tourists, whose activities, such as backpacking and sleeping outdoors or in tents, may have increased their exposure to sand fly vectors^43^. Human Immunudeficiency Virus infection also predominantly affects adults in sexually active age groups, with transmission associated with behavioral and social factors, including multiple sexual partners and unprotected sex^40^. The overlapping age distribution of ATL and HIV infection may therefore partly explain the predominance of coinfection observed among adults aged 20-60 years.

Regarding the deaths reported in the studies included in this systematic review, age range varied among men aged 50 to 60 years in South American countries. However, when compared to other countries, no records of death from ATL-HIV coinfection were found. This may be because the disease causes ulcerative lesions and partial destruction of the membranes, leading to disability rather than mortality^1^ ^,^ ^44^.

Regarding diagnostic methods, the most common test for detecting ATL was the growth culture. The culture medium used was Schneider’s Insect Medium (Sigma-Aldrich). Compared to other alternatives, Schneider’s Insect Medium is considered a simple and valuable medium for the diagnostic of cutaneous leishmaniasis^45^. Montenegro intradermal reaction was also used, which, in addition to being one of the detection tests recommended by the Brazilian Ministry of Health, induces, through intradermal injection of *Leishmania* antigens, an adaptive immune response in individuals who had already been infected with *Leishmania* ^46^.

Regarding *Leishmania* species, *L. (Viannia) braziliensis* was the most frequently identified species in the studies, followed by *L. (Leishmania) amazonensis* and *L. (Viannia) guyanensis*, given that these are the most important species of the genus and are widely distributed in South America^1^. This is because the subgenus *Viannia* has a wide distribution because of a significant genetic diversity, which may explain its adaptation in different places in Brazil^47^.

Regarding symptoms, fever was the most frequently reported manifestation among the studies included in this systematic review. However, this finding should be interpreted with caution, because fever may reflect concomitant infections, such as visceral leishmaniasis, which are frequently observed among people living with HIV, or may be influenced by reporting bias, given that, in some studies, fever was based on patient self-report rather than objectively confirmed clinical measurements. Furthermore, fever is a well-recognized infusion-related adverse event associated with amphotericin B therapy, particularly during or shortly after intravenous administration, and may therefore have contributed to the frequency of this symptom reported across the included studies^48^. Regarding diarrhea, it has been observed that the small intestine can be compromised in HIV infection due to poor nutrient absorption and the adverse effects of antiretroviral drugs^49^. In terms of cough and dysphagia, ATL, especially in its mucosal form, can affect the throat, which could explain the higher frequency of these symptoms^1^. Headache is another symptom that can occur at different times during HIV infection and may be related to several factors, such as opportunistic infections and IRIS (immune reconstitution inflammatory syndrome)^50^.

Regarding treatment, 18 studies presented Glucantime (meglumine antimoniate) as a first-line drug for ATL, associated, in some cases, with other therapies. In situations where antimoniate is contraindicated due to adverse effects, such as gastrointestinal changes and mild to moderate headache, the use of amphotericin B is recommended^51^.

For the treatment of HIV, studies cited zidovudine (AZT) + lamivudine (3TC), recommended antiretroviral drugs that, when combined, help inhibit virus replication and reduce the presence of viral RNA in plasma^52^. One of the case report studies presented only the drug amphotericin B deoxycholate^32^ a drug of choice for the treatment of *Leishmania*-HIV coinfection in the ATL. This treatment is similar to those adopted by other countries around the world, such as Thailand, where a 47-year-old woman was treated with this medication and showed improvement in mucosal nodules and skin lesions^53^.

Regarding opportunistic infections, the most frequent one reported in the included studies was toxoplasmosis, caused by the *Toxoplasma gondii* parasite, most often leading to cerebral toxoplasmosis (toxoplasmosis encephalitis) in individuals with HIV who are already severely immunocompromised. This may occur due to the reactivation of latent tissue cysts from a past infection^54^. Candidiasis, the second most prevalent opportunistic infection described in the included studies, usually appears due to the patient's immunosuppression^54^. Tuberculosis was the third most frequent opportunistic infection in the studies analyzed. This can be explained by the fact that tuberculosis can occur at any CD4^+^ T cell count level, because in immunosuppressed individuals, the disease can become severe and systemic, with rapid progression to sepsis^54^. Regarding pneumonia, it can appear as an opportunistic infection, as it is believed to occur in people who do not know their serological status or are not receiving continuous treatment for HIV, contributing to the pathogenesis of pneumonia^54^. 

Of the countries that presented studies on ATL-HIV coinfection in South America, Brazil had the highest number of studies in the period analyzed. One study found that the country had a high number of cases of ATL and HIV/AIDS between 2000 and 2006, reaching a percentage of 43% for ML and 20% for DL, which differs from Southern Europe, which had 0.4% for the former and 4% for the latter in the same period^55^. Other South American countries, including Argentina, Bolivia, Colombia, Ecuador, Paraguay, and Peru, have also presented studies addressing coinfection, given the simultaneous transmission of ATL and HIV/AIDS in these countries, in addition to factors such as relative humidity, precipitation, and temperature, which are conducive to vector reproduction^56^. For Ecuador, Paraguay, and Peru, the analysis of clinical evolution was limited by the absence of data, highlighting a problem in the epidemiological surveillance systems of these countries^1^.

This systematic review included important data on the clinical and epidemiological aspects of ATL and HIV coinfection in South American countries, but it has limitations. Some information on sociodemographic characteristics and clinical and diagnostic methods was missing from the studies included in the research. Furthermore, the low number of reports in the literature on deaths from coinfection with ATL and HIV/AIDS is noteworthy.

The findings of the systematic review followed the PRISMA guidelines and are directly related to the search strategies used as an alternative to reduce selection bias. In addition, it is worth noting that the quality assessment of systematic review studies is important because it indicates the reliability of the review results. Twenty-one studies were classified as moderate. The present study has limitations that should be considered when interpreting the data. Given the results provided by the studies included in this systematic review, it is suggested that future studies address more complete information on the epidemiological, clinical, and diagnostic methods of ATL and HIV/AIDS coinfection. In addition, given the interaction between HIV and ATL, there is a need for joint surveillance to improve the clinical management of patients.

## CONCLUSION

Exposure to ATL and coinfection with HIV mainly affects men, presumably because they are more exposed to rural and outdoor activities. The 20-60 age group, which is the most affected because it is in its productive phase, appears to be more exposed to vectors due to respective working environments. In addition, for HIV/AIDS, this age group may have been the most affected by behavioral and social factors, such as low educational attainment. In this sense, joint surveillance, access to and education about health, and further studies on coinfection should be reinforced to achieve, in addition to improving the health of individuals, a better understanding of the dynamics of the association of these diseases in South American countries.

## Data Availability

Data-in-article: Research data is available in the body of the article.
