# Supplementary material for: Clinical and epidemiological aspects of coinfection with American tegumentary leishmaniasis and HIV/AIDS in South American countries: a systematic review
Source: Rev Soc Bras Med Trop. 2026 Oct 16;59:e0128-2026. doi: 10.1590/0037-8682-0128-2026 (PMC13674198; doi:10.1590/0037-8682-0128-2026)
Supplement: Supplementary file 1 [file 1678-9849-rsbmt-59-e0128-2026-md1.pdf]

Revista da Sociedade Brasileira de Medicina Tropical
Journal of the Brazilian Society of Tropical Medicine

Supplementary Material Table 1 - Assessment of the risk of bias of the case report studies included in the review.

Questions/ Author	Were patient's demographic characteristics clearly described?	Was the patient's history clearly described and presented as a timeline?	Was the current clinical condition of the patient on presentation clearly described?	Were diagnostic tests or assessment methods and the results clearly described?	Was the intervention(s) or treatment procedure(s) clearly described?	Was the post-intervention clinical condition clearly described?	Were adverse events (harms) or unanticipated events identified and described?	Does the case report provide takeaway lessons?	Risk of bias
**Coura *et al.*[8]**	No	Yes	Yes	Yes	No	Yes	Yes	No	**Medium**
**Da-Cruz *et al.*[9]**	No	No	Yes	Yes	No	No	No	Yes	**High**
**Machado *et al.*[10]**	No	Não	Yes	Yes	No	Yes	Yes	Yes	**Medium**
**Souza *et al.*[11]**	No	Yes	Yes	Yes	Yes	Yes	No	Yes	**Medium**
**Rosatelli *et al.*[12]**	No	Yes	Yes	Yes	Yes	Yes	Yes	Yes	**Low**
**Da-Cruz *et al.*[13]**	No	Yes	Yes	No	Yes	Yes	Yes	No	**Medium**
**Romero *et al.*[14]**	No	Yes	Yes	No	Yes	Yes	Yes	Yes	**Medium**
**Pérez *et al.*[15]**	No	Yes	Yes	Yes	Yes	Yes	No	No	**Medium**
**Chrusciak-Talhari *et al.*[17]**	No	Yes	Yes	Yes	Yes	Yes	No	No	**Medium**

**Torrico *et al*.**[18]	No	Yes	Yes	No	No	Yes	No	Yes	**Medium**
**Castellano *et al*.**[20]	No	Yes	Yes	Yes	No	No	No	Yes	**Medium**
**Hozannah *et al*.**[21]	No	No	Yes	Yes	Yes	Yes	No	No	**Medium**
**Montenegro-Idrogo *et al*.**[22]	No	Yes	Yes	Yes	Yes	Yes	No	Yes	**Medium**
**Calvopiña *et al*.**[23]	No	Yes	Yes	Yes	Yes	Yes	Yes	Yes	**Low**
**Soares *et al*.**[25]	No	Yes	Yes	Yes	Yes	Yes	No	Yes	**Medium**
**Araújo *et al*.**[26]	No	Yes	Yes	Yes	Yes	Yes	Yes	Yes	**Low**
**Amato *et al*.**[27]	No	Yes	No	Yes	Yes	No	No	Yes	**Medium**
**Laverde-Sudupe *et al*.**[28]	No	Yes	Yes	No	Yes	Yes	Yes	Yes	**Medium**
**Da Silva *et al*.**[29]	No	Yes	Yes	Yes	Yes	Yes	No	Yes	**Medium**
**Parmentier *et al*.**[31]	No	Yes	Yes	Yes	Yes	Yes	No	Yes	**Medium**
**Durães *et al*.**[32]	No	Yes	Yes	No	Yes	Yes	Yes	Yes	**Medium**
