# Supplementary material for: Clinical and epidemiological aspects of coinfection with American tegumentary leishmaniasis and HIV/AIDS in South American countries: a systematic review
Source: Rev Soc Bras Med Trop. 2026 Oct 16;59:e0128-2026. doi: 10.1590/0037-8682-0128-2026 (PMC13674198; doi:10.1590/0037-8682-0128-2026)
Supplement: Supplementary file 2 [file 1678-9849-rsbmt-59-e0128-2026-md2.pdf]

Supplementary Material Table 2 - Assessment of the risk of bias of the cross-sectional studies included in the review.

Questions/ Author	Were the criteria for inclusion in the sample clearly defined?	Were the study subjects and the setting described in detail?	Was the exposure measured in a valid and reliable way?	Were objective, standard criteria used for measurement of the condition?	Were confounding factors identified?	Were strategies to deal with confounding factors stated?	Were the outcomes measured in a valid and reliable way?	Was appropriate statistical analysis used?	Risk of bias
**Guimarães *et al*.[30]**	Yes	Yes	Not applicable	Yes	Yes	No	Yes	No	**Medium**
**De Oliveira *et al*.[33]**	Yes	Yes	Yes	Yes	Yes	No	Yes	Yes	**Low**
**Rodrigues *et al.*[36]**	Yes	Yes	Yes	Yes	No	No	Yes	Yes	**Medium**
**Oliveira *et al*.[37]**	Yes	No	Yes	Yes	Yes	No	Yes	Yes	**Medium**
