# Supplementary material for: Clinical and epidemiological aspects of coinfection with American tegumentary leishmaniasis and HIV/AIDS in South American countries: a systematic review
Source: Rev Soc Bras Med Trop. 2026 Oct 16;59:e0128-2026. doi: 10.1590/0037-8682-0128-2026 (PMC13674198; doi:10.1590/0037-8682-0128-2026)
Supplement: Supplementary file 3 [file 1678-9849-rsbmt-59-e0128-2026-md3.pdf]

Supplementary Material Table 3 - Assessment of the risk of bias of the case series studies included in the review.

Questions/Author	Were there clear criteria for inclusion in the case series?	Was the condition measured in a standard, reliable way for all participants included in the case series?	Were valid methods used for identification of the condition for all participants included in the case series?	Did the case series have consecutive inclusion of participants?	Did the case series have complete inclusion of participants?	Was there clear reporting of the demographics of the participants in the study?	Was there clear reporting of clinical information of the participants?	Were the outcomes or follow up results of cases clearly reported?	Was there clear reporting of the presenting site(s)/clinic(s) demographic information?	Was statistical analysis appropriate?	Risk of bias
**Lyra *et al.*[24]**	Yes	Yes	Yes	Yes	Yes	Yes	Yes	No	No	No	**Low**
**Guerra *et al.*[35]**	Yes	Yes	Yes	Yes	Yes	Yes	Yes	Yes	No	No	**Low**
**Gois *et al.*[38]**	Yes	Yes	Yes	Yes	Yes	Yes	Yes	Yes	No	Sim	**Low**
