# Supplementary material for: Clinical and epidemiological aspects of coinfection with American tegumentary leishmaniasis and HIV/AIDS in South American countries: a systematic review
Source: Rev Soc Bras Med Trop. 2026 Oct 16;59:e0128-2026. doi: 10.1590/0037-8682-0128-2026 (PMC13674198; doi:10.1590/0037-8682-0128-2026)
Supplement: Supplementary file 4 [file 1678-9849-rsbmt-59-e0128-2026-md4.pdf]

Revista da Sociedade Brasileira de Medicina Tropical
Journal of the Brazilian Society of Tropical Medicine

Supplementary Material Table 4 - Assessment of the risk of bias of the case-control studies included in the review.

Questions/Author	Were the groups comparable other than the presence of disease in cases or the absence of disease in controls?	Were cases and controls matched appropriately?	Were the same criteria used for identification of cases and controls?	Was exposure measured in a standard, valid and reliable way?	Was exposure measured in the same way for cases and controls?	Were confounding factors identified?	Were strategies to deal with confounding factors stated?	Were outcomes assessed in a standard, valid and reliable way for cases and controls?	Was the exposure period of interest long enough to be meaningful?	Was appropriate statistical analysis used?	Risk of bias
**Soares *et al*.**[16]	No	Yes	Yes	Yes	No	Yes	Uncertain	Yes	Yes	Yes	**Low**
**Santos-Oliveira *et al*.**[19]	No	Yes	Yes	Yes	Yes	No	No	Yes	Yes	Yes	**Low**
**Couppie et al.** [39]	Yes	Yes	Yes	Yes	Yes	No	No	Yes	Yes	Yes	**Low**
