# Supplementary material for: Clinical and epidemiological aspects of coinfection with American tegumentary leishmaniasis and HIV/AIDS in South American countries: a systematic review
Source: Rev Soc Bras Med Trop. 2026 Oct 16;59:e0128-2026. doi: 10.1590/0037-8682-0128-2026 (PMC13674198; doi:10.1590/0037-8682-0128-2026)
Supplement: Supplementary file 5 [file 1678-9849-rsbmt-59-e0128-2026-md5.pdf]

Supplementary Material Table 5 - Assessment of the risk of bias of the cohort studies included in the review.

Question/Author	Were the two groups similar and recruited from the same population?	Were the exposures measured similarly to assign people to both exposed and unexposed groups?	Was the exposure measured in a valid and reliable way?	Were confounding factors identified?	Were strategies to deal with confounding factors stated?	Were the groups/participants free of the outcome at the start of the study (or at the moment of exposure)?	Were the outcomes measured in a valid and reliable way?	Was the follow up time reported and sufficient to be long enough for outcomes to occur?	Was follow up complete, and if not, were the reasons to loss to follow up described and explored?	Were strategies to address incomplete follow up utilized?	Was appropriate statistical analysis used?	Risk of bias
**Amato *et al.*[34]**	Yes	Yes	Yes	Yes	Yes	Yes	Yes	Yes	Uncertain	No	Yes	**Low**
